# Epitranscriptomic m^6^A modifications during reactivation of HIV-1 latency in CD4^+^ T cells

**DOI:** 10.1128/mbio.02214-24

**Published:** 2024-10-07

**Authors:** Tarun Mishra, Stacia Phillips, Yutao Zhao, Bethany Wilms, Chuan He, Li Wu

**Affiliations:** 1 Department of Microbiology and Immunology, Carver College of Medicine, The University of Iowa, Iowa City, Iowa, USA; 2 Department of Chemistry, Department of Biochemistry and Molecular Biology, Institute for Biophysical Dynamics, University of Chicago, Chicago, Illinois, USA; 3 Howard Hughes Medical Institute, University of Chicago, Chicago, Illinois, USA; Dana-Farber Cancer Institute, Boston, Massachusetts, USA

**Keywords:** RNA m^6^A modification, HIV-1 latency reversal, J-Lat cells, primary CD4^+^T cells, *POLR3H*, *TUG1*

## Abstract

Despite effective antiretroviral therapy reducing HIV-1 viral loads to undetectable levels, the presence of latently infected CD4^+^ T cells poses a major barrier to HIV-1 cure. *N*^6^-methyladenosine (m^6^A) modification of viral and cellular RNA has a functional role in regulating HIV-1 infection. m^6^A modification of HIV-1 RNA can affect its stability, translation, and splicing in cells and suppresses type-I interferon induction in macrophages. However, the function of m^6^A modification in regulating HIV-1 latency reactivation remains unknown. We used the Jurkat T cell line-derived HIV-1 latency model (J-Lat cells) to investigate changes in m^6^A levels of cellular RNA in response to latency reversal. We observed a significant increase in m^6^A levels of total cellular RNA upon reactivation of latent HIV-1 in J-Lat cells. This increase in m^6^A levels was transient and returned to steady-state levels despite continued high levels of viral gene expression in reactivated cells compared to control cells. Upregulation of m^6^A levels occurred without significant changes in the protein expression of m^6^A writers or erasers that add or remove m^6^A, respectively. Knockdown of m^6^A writers in J-Lat cells significantly reduced HIV-1 reactivation. Treatment with an m^6^A writer inhibitor reduced cellular RNA m^6^A levels, along with a reduction in HIV-1 reactivation. Furthermore, using m^6^A-specific sequencing, we identified cellular RNAs that are differentially m^6^A-modified during HIV-1 reactivation in J-Lat cells. Knockdown of identified m^6^A-modified RNA validates these results with an established primary CD4^+^ T cell model of HIV-1 latency. These results show the importance of m^6^A RNA modification in HIV-1 latency reversal.

**IMPORTANCE:**

RNA m^6^A modification is important for regulating gene expression and innate immune responses to HIV-1 infection. However, the functional significance of m^6^A modification during HIV-1 latency reactivation is unknown. To address this important question, in this study, we used established cellular models of HIV-1 latency, m^6^A-specific sequencing at single-base resolution, and functional assays. We demonstrate that HIV-1 latency reversal leads to increased levels of cellular m^6^A modification, correlates with cellular m^6^A levels, and is dependent on the catalytic activity of the m^6^A methyltransferase enzyme. We also identified cellular genes that are differentially m^6^A-modified during HIV-1 reactivation, as well as the sites of m^6^A within HIV-1 RNA. Our novel findings point toward a significant role for m^6^A modification in HIV-1 latency reversal.

## INTRODUCTION

Human immunodeficiency virus type 1 (HIV-1) infection remains a formidable global health challenge despite advancements in antiretroviral therapy (ART). A major obstacle to achieving a cure for HIV-1 infection is the persistence of latent viral reservoirs within long-lived CD4^+^ T cells (1). These latent reservoirs harbor transcriptionally silent HIV-1 proviral DNA, evading immune detection and conventional ART regimens (2). Various mechanisms contribute to HIV-1 latency, including host transcription factor sequestration in the cytoplasm and transcriptional repression (3). Current strategies toward HIV-1 cure include the establishment of a permanent state of latency (block and lock) or inducing reactivation from latency so that infected cells can be killed by the immune system (shock and kill) (4–6).

*N*^6^-methyladenosine (m^6^A) is the most abundant internal modification in eukaryotic RNA and is critical for regulating cellular gene expression and physiological function of RNA (7, 8). The dynamic regulation of m^6^A modification involves two groups of proteins known as writers and erasers, which respectively add or remove m^6^A marks (7, 8). Methyltransferase-like 3 (METTL3), methyltransferase-like 14 (METTL14), and Wilms’ tumor 1-associated protein (WTAP) form the m^6^A writer complex and catalyze the addition of a methyl group to the *N*^6^ position of adenosine within mRNA (9, 10). Conversely, fat mass and obesity-associated protein (FTO) and AlkB family member 5 (ALKBH5) function as erasers, removing m^6^A marks and thereby influencing mRNA fate and cellular functions (11, 12).

Manipulation of m^6^A modification or its recognition by m^6^A-binding proteins regulates various aspects of HIV-1 infection, including gene expression, splicing, translation, genome packaging, and evasion of innate immune responses (13). In addition, HIV-1 infection induces an increase in the level of cellular RNA m^6^A (14–17). We previously demonstrated that HIV-1 upregulates m^6^A levels in activated primary CD4^+^ T cells independently of viral replication (15). We recently reported that cellular RNA m^6^A levels in peripheral blood mononuclear cells (PBMCs) of HIV-1-infected individuals receiving suppressive ART are reduced compared to those with viremia (18). However, the functional role of cellular RNA m^6^A modification in HIV-1 latency reversal is not understood. Investigating changes in specific cellular RNA m^6^A modifications during HIV-1 latency reversal is important for understanding how m^6^A modifications impact cellular RNA dynamics and function.

In this study, we explored the impact of HIV-1 latency reversal on cellular RNA m^6^A levels and defined the m^6^A epitranscriptome in reactivated cells at single-base resolution. We used well-defined J-Lat cell line model (19) and primary central memory CD4^+^ T cell (T_CM_) model of HIV-1 latency (20). We show that HIV-1 latency reversal enhances cellular RNA m^6^A levels. m^6^A writer expression or catalytic activity was positively correlated with reactivation levels. Furthermore, we observed altered m^6^A modification in a specific set of cellular transcripts upon reactivation of J-Lat cells compared to controls. Finally, we demonstrated that depletion of select differentially m^6^A-modified cellular RNAs impaired HIV-1 latency reversal upon reactivation in both J-Lat and latently infected primary T_CM_ cells. These findings suggest that differential m^6^A modification is an important regulatory mechanism influencing HIV-1 latency reversal.

## RESULTS

### HIV-1 latency reversal in CD4^+^ T cells enhances cellular RNA m^6^A levels

We and others have reported that HIV-1 infection of various cell types leads to an increase in the abundance of m^6^A in total cellular RNA (14–17). We sought to determine whether the levels of m^6^A modification also increase in cellular RNA after the reactivation of latent HIV-1 gene expression. We used J-Lat cells, which are clonally selected Jurkat cell lines harboring a single copy of HIV-1 proviral DNA with a premature stop codon in envelope (*env*) and a green fluorescent protein (GFP) reporter in place of *nef* (19). Under steady-state conditions, these cells do not express detectable GFP, but upon treatment with latency reversal agents (LRA), they express HIV-1 genes and HIV-1 promoter-driven GFP reporter (19). Treatment of J-Lat cells with phorbol 12-myristate 13-acetate (PMA) and ionomycin (P + I) as a potent LRA efficiently activates HIV-1 gene and GFP expression (21).

**Fig 1:**
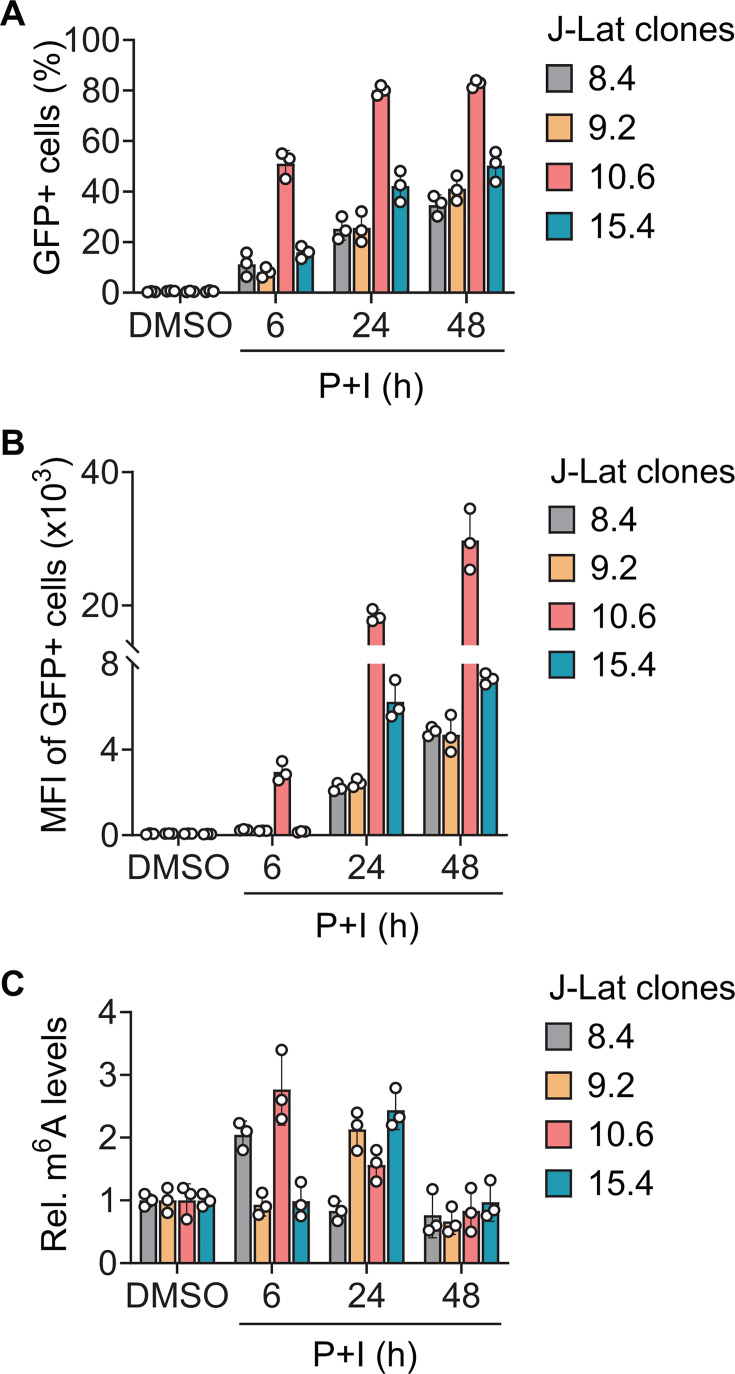
HIV-1 latency reversal enhances cellular RNA m^6^A levels. J-Lat cells were treated with DMSO as a negative control or PMA and ionomycin (P + I) for 6, 24, or 48 h to induce reactivation of HIV-1 gene expression. (**A**) GFP-positive cells and (**B**) MFI were measured by flow cytometry. (**C**) Total cellular RNA m^6^A levels were measured by m^6^A dot blot after treatment with DMSO (6 h) or P + I at the indicated time points. Results are shown as mean ± SD from three independent experiments.

We first screened different clones of J-Lat cells to determine the level and kinetics of GFP expression upon treatment with P + I for 6, 24, or 48 h. Treatment of J-Lat cells with dimethyl sulfoxide (DMSO) as a vehicle control did not induce any detectable GFP expression (Fig. 1A and B). We found that J-Lat clone 10.6 exhibited the highest levels of GFP expression, based on both the percentage and mean fluorescence intensity (MFI) of positive cells measured by flow cytometry (Fig. 1A and B). The kinetics of GFP expression after P + I treatment showed approximately 50% of GFP expression after only 6 h, which further increases and plateaus at 24 and 48 h of treatment (Fig. 1A). J-Lat cell clones 8.4, 9.2, and 15.4 showed similar kinetics of GFP expression, while the percentage of positive cells was lower than clone 10.6 at all tested time-points (Fig. 1A and B).

We next asked whether HIV-1 reactivation influences cellular RNA m^6^A levels. J-Lat cells were treated with P + I for 6, 24, or 48 h, and m^6^A levels were measured in total RNA using m^6^A-specific dot blot (22). P + I treatment of all J-Lat clones resulted in a transient increase in cellular RNA m^6^A levels compared to DMSO-treated control cells (Fig. 1C). However, the kinetics of m^6^A upregulation were different among J-Lat clones, with clones 8.4 and 10.6 showing peak upregulation at 6 h and clones 9.2 and 15.4 showing elevated m^6^A at 24 h post-reactivation (Fig. 1C). For all clones, the levels of m^6^A returned to that of control cells by 48 h, despite sustained GFP expression (Fig. 1A and B).

Our goal in this study was to identify epitranscriptomic changes that occur in temporal association with HIV-1 latency reactivation. We reasoned that using the shortest possible P + I treatment time would most closely mimic the state of reactivation. Based on the results shown in Fig. 1, we chose J-Lat clone 10.6 and 6 h of P + I treatment for our subsequent studies.

### METTL3 supports efficient HIV-1 latency reversal

**Fig 2:**
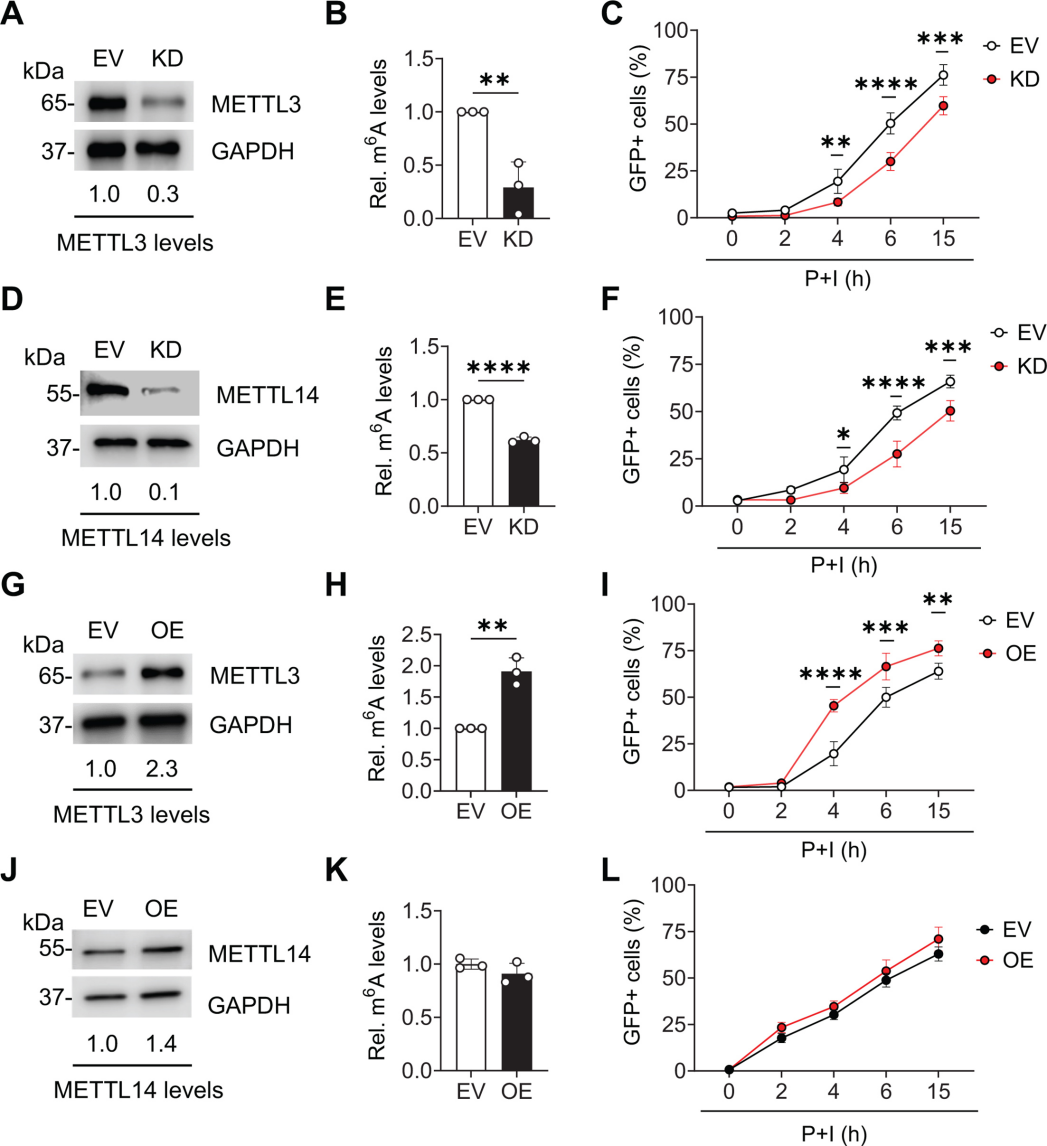
Modulation of the m^6^A methyltransferase complex influences HIV-1 reactivation. J-Lat 10.6 cells were transduced with lentiviral vectors for stable KD or OE of the methyltransferase subunit METTL3 or METTL14. EVs were used to generate control cell lines. (**A and D**) KD and (**G and J**) OE of the target proteins were confirmed by immunoblotting. Densitometry values were normalized to glyceraldehyde-3-phosphate dehydrogenase (GAPDH) levels. Cellular RNA m^6^A levels from (**B**) METTL3 KD, (**E**) METTL14 KD, (**H**) METTL3 OE, and (**K**) METTL14 OE cell lines were determined using m^6^A-specific enzyme-linked immunosorbent assay (ELISA). Cells were treated with P + I, and GFP-positive cells were quantified by flow cytometry at the indicated timepoints. (**C**) METTL3 KD, (**F**) METTL14 KD, (**I**) METTL3 OE, and (**L**) METTL14 OE. Results are shown as mean ± SD from three independent experiments. Statistical analysis was performed using unpaired *t*-test (B, E, H, and K) or two-way analysis of variance (ANOVA; C, F, I, and L). *P* values are indicated by *P < 0.05, **P < 0.01, ***P < 0.001, and ****P < 0.0001.

We next sought to determine whether cellular RNA m^6^A levels can affect the efficiency of latent HIV-1 reactivation. To manipulate the levels of cellular RNA m^6^A, we generated several stable cell lines to either knockdown (KD) or overexpress (OE) METTL3 or METTL14, the protein subunits of the catalytic core of the m^6^A methyltransferase complex. METTL3 or METTL14 depleted J-Lat 10.6 cells were generated by lentiviral transduction with vectors expressing Cas9 and gene-specific single guide RNA (sgRNA), followed by antibiotic selection. We were unable to generate a clonal population of cells that contained a complete knockout of METTL3 or METTL14. Therefore, our experiments were performed with clones exhibiting significant KD of endogenous gene expression as determined by immunoblotting (Fig. 2A and D). J-Lat 10.6 cells transduced with empty vector (EV) were used as controls. Stable OE of METTL3 and METTL14 was achieved by transduction with Flag-METTL3 or Flag-METTL14 lentiviral vectors followed by antibiotic selection. OE of METTL3 and METTL14 was confirmed by immunoblotting (Fig. 2G and J).

The cellular RNA m^6^A levels in METTL3 or METTL14 KD and OE cells were assessed by m^6^A enzyme-linked immunosorbent assay (ELISA) (23). KD of either METTL3 or METTL14 in J-Lat 10.6 cells significantly reduced cellular RNA m^6^A levels (Fig. 2B and E). In contrast, when METTL3 was overexpressed, we observed a significant increase in cellular RNA m^6^A levels (Fig. 2H). OE of METTL14 did not affect cellular RNA m^6^A levels (Fig. 2K). These results confirm that METTL3 is the subunit containing catalytic activity and suggest that the levels of METTL3 are limiting for active methyltransferase complex formation.

We next wanted to determine whether cellular m^6^A is required for the efficient reactivation of HIV-1 gene expression in latently infected cells. Control, METTL3 KD and OE, and METTL14 KD and OE cells were treated with P + I to reactivate HIV-1 gene expression. Cells were collected for the measurement of GFP expression at the indicated times post-reactivation (Fig. 2C, F, I and L). The results showed reduced HIV-1 reactivation in METTL3 and METTL14 KD cells compared to control cells (Fig. 2C and F), whereas METTL3 OE cells showed higher levels of reactivation (Fig. 2I). METTL14 OE had no effect on reactivation compared to control cells (Fig. 2L). These data show that the levels of m^6^A writer complex and, therefore, m^6^A modification, correlate with the levels of HIV-1 gene expression upon latency reactivation.

### METTL3 activity inhibitor reduces HIV-1 reactivation

**Fig 3:**
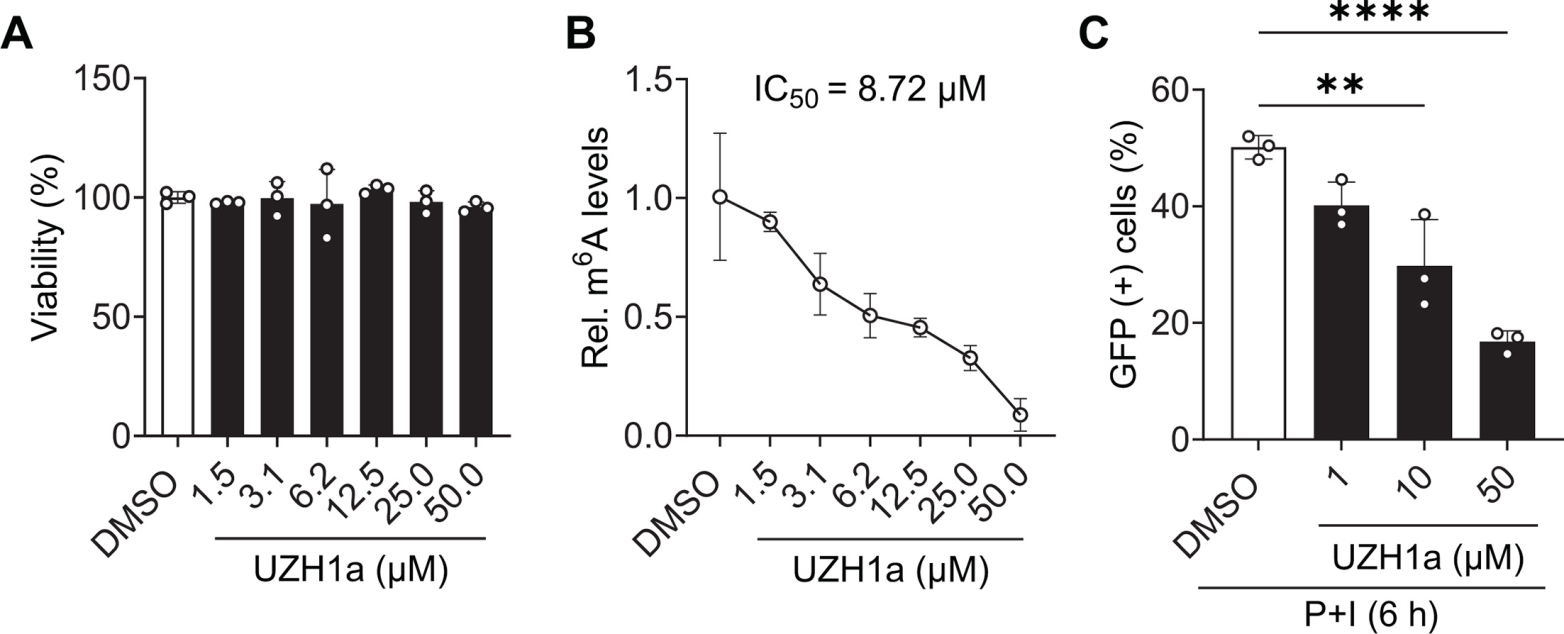
Inhibition of METTL3 activity reduces HIV-1 latency reversal. J-Lat 10.6 cells were treated with DMSO or the METTL3 inhibitor UZH1a for 48 h prior to treatment with P + I for 6 h. (**A**) Cell viability was measured by the MTT [3-(4,5-dimethylthiazol-2-yl)-2,5-diphenyltetrazolium bromide] assay after treatment with UZH1a at the indicated concentrations. (**B**) Relative poly(A) RNA m^6^A levels were measured by ELISA. UZH1a inhibits METTL3 activity with IC_50_ = 8.73 µM. (**C**) GFP-positive cells were measured by flow cytometry after treatment with P + I. Results are shown as mean ± SD from three independent experiments. Statistical analysis was performed using one-way ANOVA. **P < 0.01 and ****P < 0.0001.

Since METTL3 is the only known protein with methyltransferase activity in mammalian cells and its KD and OE influence HIV-1 reactivation, we hypothesized that inhibition of METTL3 catalytic activity will inhibit HIV-1 reactivation. To test this, we first treated J-Lat 10.6 cells with variable concentrations of the METTL3 inhibitor UZH1a (24) for 48 h and then assessed cell viability (25). The results showed comparable cell viability over the range of UZH1a concentrations from 1.5 to 50 µM (Fig. 3A). Next, we determined the dose-dependent response of UZH1a treatment on cellular RNA m^6^A levels in J-Lat 10.6 cells. Cellular RNA m^6^A levels were assessed using m^6^A ELISA after 48 h of UZH1a treatment. UZH1a treatment showed a dose-dependent reduction in m^6^A levels (Fig. 3B), with half maximal inhibitory concentration (IC_50_) of 8.72 µM. Subsequently, to determine the effect of METTL3 inhibition on HIV-1 reactivation, J-Lat 10.6 cells were treated with UZH1a at 1, 10, or 50 µM for 48 h followed by treatment with P + I. HIV-1 reactivation was assessed by measuring GFP expression. We observed a direct correlation between the reduction of cellular RNA m^6^A levels and the levels of HIV-1 reactivation (Fig. 3C). These results suggest that METTL3 activity is required for efficient HIV-1 latency reversal in J-Lat 10.6 cells.

### Activation of uninfected CD4^+^ T cell lines does not affect cellular RNA m^6^A level

**Fig 4:**
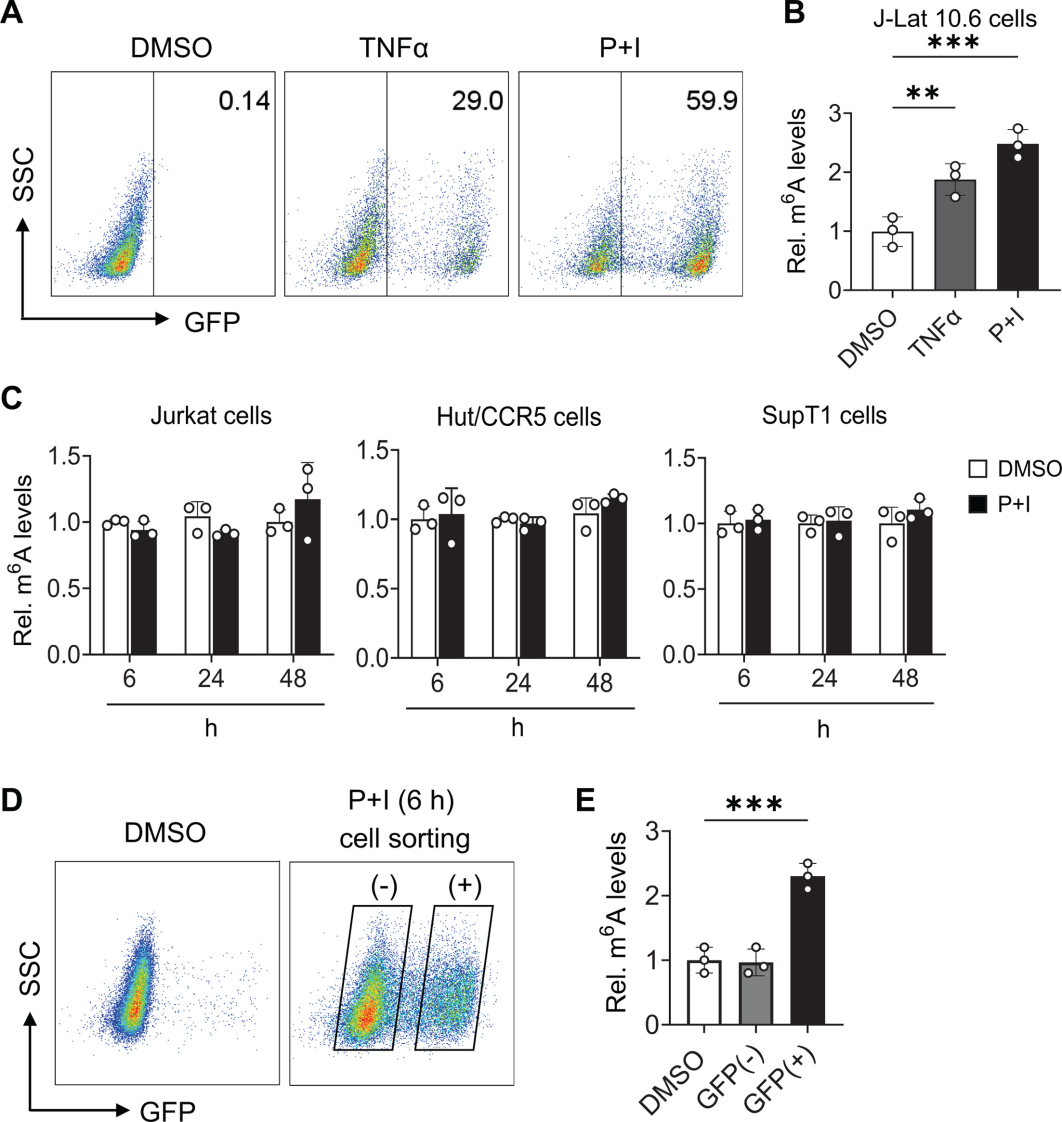
Treatment of uninfected CD4^+^ T cell lines with LRA does not affect cellular RNA m^6^A level. (**A**) J-Lat 10.6 cells were treated with DMSO, TNFα, or P + I for 6 h, and GFP-positive cells were quantified by flow cytometry. SSC, side scatter. (**B**) Relative cellular RNA m^6^A levels in cells from (**A**) were measured by dot blot. (**C**) CD4^+^ T cells lines Jurkat, Hut/CCR5, and SupT1 were treated with DMSO or P + I, and relative cellular RNA m^6^A levels were measured by dot blot. (**D**) J-Lat 10.6 cells were treated with DMSO or P + I for 6 h and then sorted based on GFP positivity. Flow cytometry plot shows the gating strategy for sorting. (**E**) Total cellular RNA m^6^A levels of GFP (−) and (+) cells were determined using dot blot. Results are shown as mean ± SD from three independent experiments. Statistical analysis was performed using one-way ANOVA. **P < 0.01 and ***P < 0.001.

We next wanted to confirm that the reactivation of HIV-1, and not P + I treatment, in J-Lat 10.6 cells is the reason for the increase in cellular RNA m^6^A levels. First, we treated J-Lat 10.6 cells with tumor necrosis factor alpha (TNFα), which is an alternative LRA to activate NF-kB, HIV-1 gene, and GFP expression (19). We found that while the levels of reactivation were lower with TNFα than with P + I, RNA m^6^A levels of TNFα-treated cells were still significantly higher than that of DMSO-treated cells (Fig. 4A and B). Next, we treated Jurkat, Hut/CCR5, and SupT1 CD4^+^ T cell lines that do not harbor latent HIV-1 with P + I and quantified cellular RNA m^6^A levels. We did not observe any differences in m^6^A levels between DMSO- and P + I-treated cells (Fig. 4C). Furthermore, J-Lat 10.6 cells were treated with P + I followed by sorting of GFP-positive and -negative cells (Fig. 4D). RNA was harvested from DMSO-treated cells and the sorted populations of P + I treated cells, and m^6^A levels were quantified. The results showed no change in cellular RNA m^6^A levels between DMSO-treated cells and P + I-treated cells that were GFP negative (Fig. 4E). Interestingly, a 2.3-fold increase in m^6^A was observed in GFP-positive cells, which harbor reactivated HIV-1. Together, these data suggest that reactivation of HIV-1, but not T cell activation alone, is responsible for the upregulation of cellular RNA m^6^A levels.

We hypothesized that the increase in m^6^A levels could be due to changes in the expression or subcellular localization of m^6^A regulatory proteins. To address this, we performed immunoblotting for m^6^A writer and eraser proteins in lysates from GFP-positive and -negative cells after treatment with P + I. The results did not show any significant change in expression (Fig. S1A) or localization of proteins in the cytoplasm or the nucleus (Fig. S1B). These results suggest that HIV-1 reactivation increases cellular RNA m^6^A levels independently of alteration in expression or localization of m^6^A regulatory proteins.

### m^6^A-selective allyl chemical labeling and sequencing identifies transcriptomic m^6^A modifications in cellular and HIV-1 RNA

**Fig 5:**
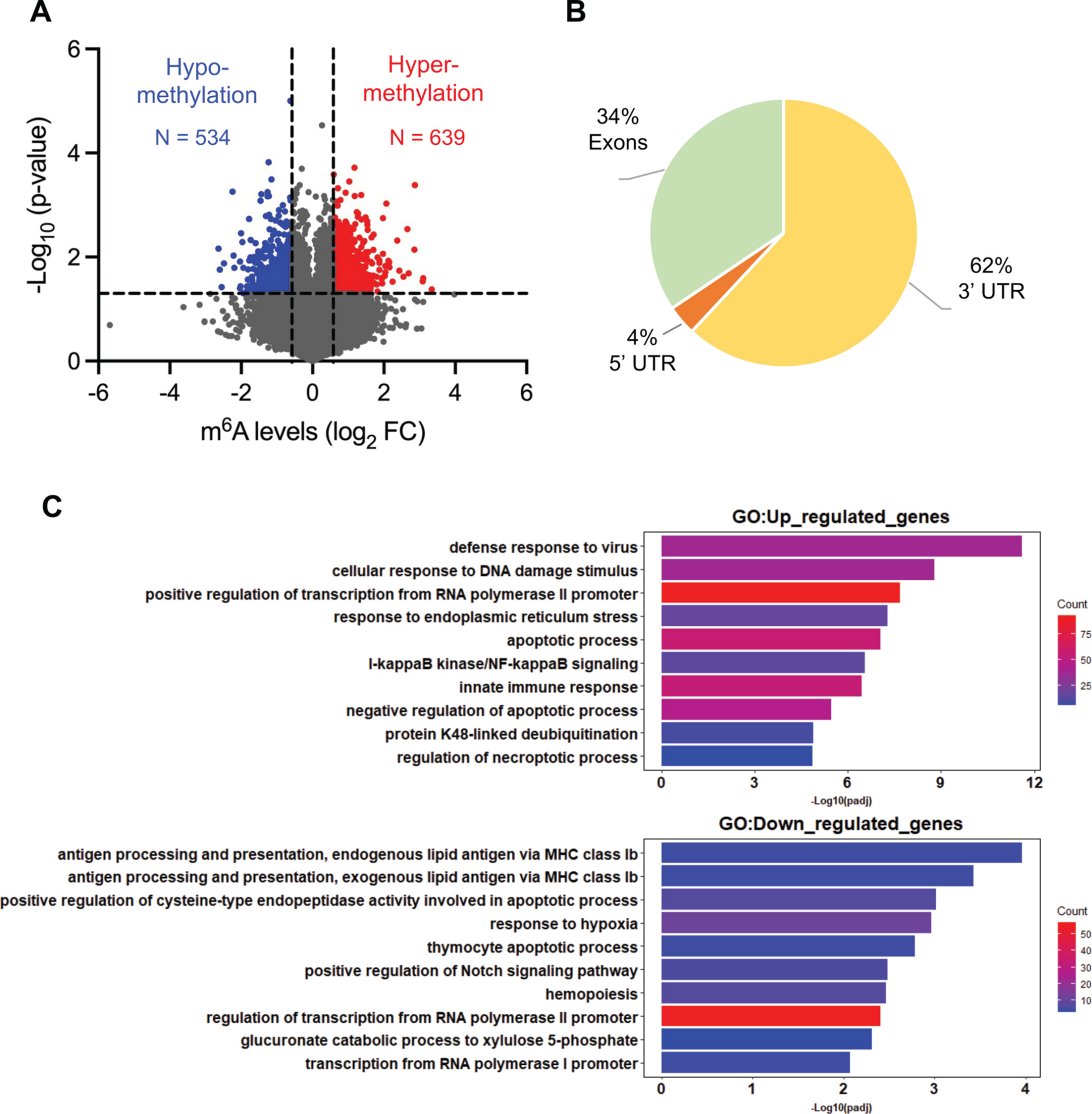
m^6^A-SAC-seq identifies cellular RNAs that are differentially m^6^A-modified upon HIV-1 reactivation. (**A**) Volcano plot representing m^6^A-hypomethylated (blue) and m^6^A-hypermethylated (red) RNA from P + I-treated cells compared to the DMSO-treated control. Transcripts that are considered differentially methylated have sites of m^6^A that are ≥1.5-fold different in reactivated cells than DMSO control, with *P* ≤ 0.01. (**B**) Distribution of m^6^A modification in different regions of cellular mRNA. Analysis was performed using DMSO and P + I samples combined (*N* = 6). (**C**) Gene ontology (GO) analysis of m^6^A-modified cellular genes in HIV-1 reactivated J-Lat 10.6 cells. The top panel of up-regulated genes represents m^6^A-hypermethylated transcripts. The bottom panel of down-regulated genes represents m^6^A-hypomethylated transcripts. Top 10 pathways and corresponding -Log_10_ *P* values in m^6^A-hypermethylated or m^6^A-hypomethylated transcripts are shown. padj, the Benjamini-Hochberg adjusted *P*-value.

Low-resolution m^6^A mapping methods, such as methylated RNA immunoprecipitation sequencing (meRIP-seq), can identify RNA transcripts that are m^6^A modified, but it is often difficult to predict which adenosine is methylated due to the presence of multiple putative m^6^A consensus motifs (13). To identify m^6^A modifications on viral and cellular RNA at single-base resolution, we utilized m^6^A-selective allyl chemical labeling and sequencing (m^6^A-SAC-seq) (26, 27). RNA from J-Lat 10.6 cells treated with either DMSO or P + I was subjected to poly(A) enrichment and then m^6^A-SAC-seq. The sequencing data were analyzed to identify transcripts that are differentially m^6^A-modified upon treatment with P + I compared to DMSO-treated controls based on statistical analysis of three biological replicates (Fig. 5A; Table S1). This analysis revealed 55,127 individual m^6^A modifications on 8,161 unique transcripts (Gene Expression Omnibus #GSE273614). We found differential m^6^A modification in response to HIV-1 reactivation in 902 unique transcripts, with 534 m^6^A sites that become hypomethylated (blue) and 639 sites that become hypermethylated (red) upon P + I treatment (Fig. 5A). Analysis of the mapped location of all m^6^A reveal that the majority of modified sites are in the 3′ UTR (62%), followed by exons (34%) and the 5′ UTR (4%; Fig. 5B). This distribution is consistent with a previous report of m^6^A mapping in cellular RNA (28).

To analyze differentially methylated cellular transcripts during HIV-1 reactivation, we performed gene ontology (GO) analysis and selected the top 10 most enriched categories in m^6^A-hypermethylated or -hypomethylated genes based on statistical significance (Fig. 5C). Among m^6^A-hypermethylated genes, the top three represented categories were defense response to virus, cellular response to DNA damage stimulus, and transcription from RNA polymerase II promoter. Notably, NF-kB signaling which is crucial for HIV-1 proviral transcription (29) was among these enriched pathways (Fig. 5C, top panel). Additionally, m^6^A- hypomethylated genes predicted top two pathways related to MHC class Ib (Fig. 5C, bottom panel), which is downregulated in HIV-1-infected cells, impairing the ability of cytotoxic T lymphocytes to lyse infected cells (30). These findings suggest that changes in the m^6^A profile of many cellular transcripts are functionally significant for HIV-1 reactivation.

**Fig 6:**
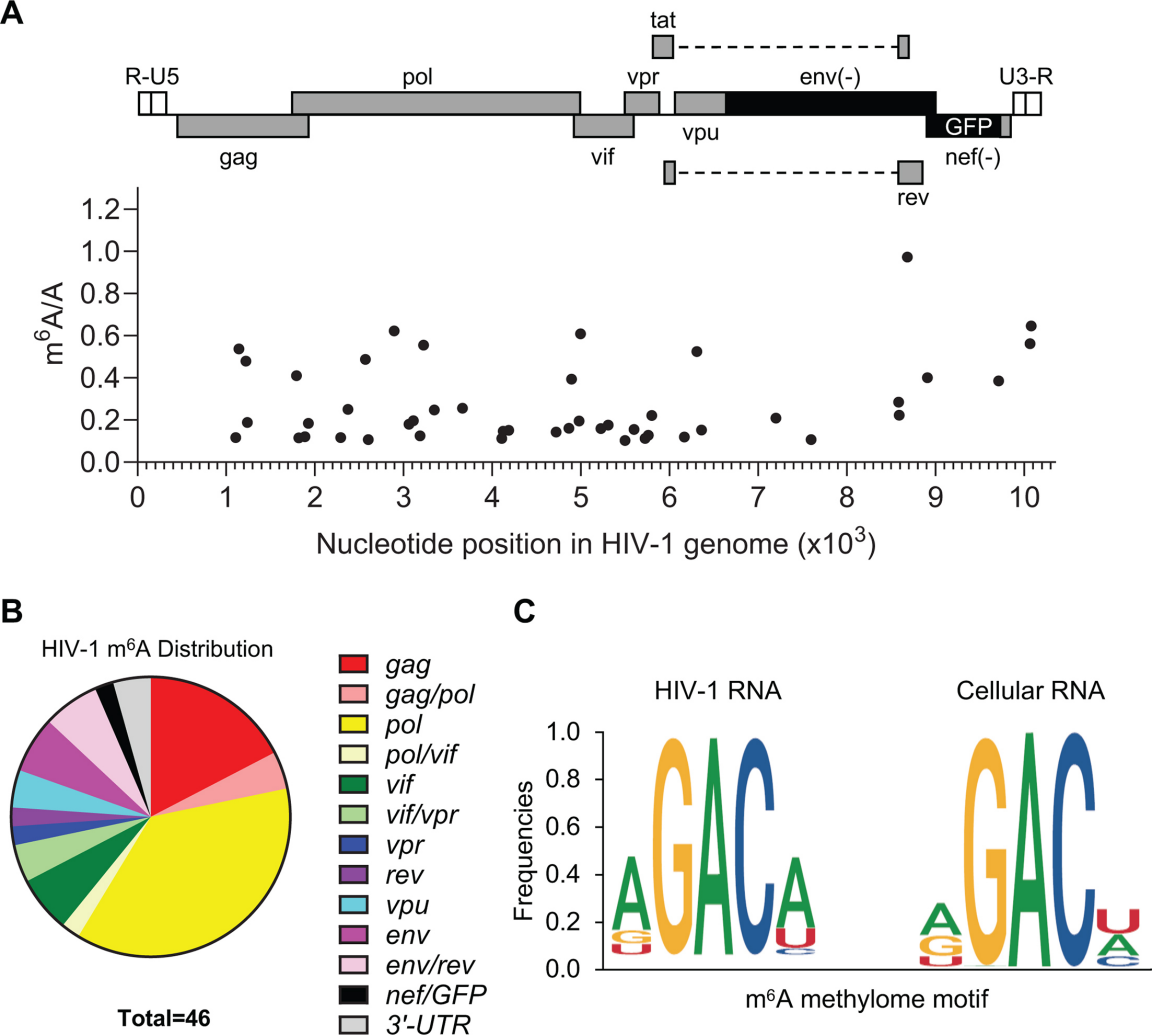
m^6^A-SAC-seq identifies novel m^6^A modification sites in HIV-1 RNA. J-Lat 10.6 cells were treated with P + I for 6 h prior to the purification of poly(A) RNA for m^6^A-SAC-seq. (**A**) HIV-1 RNA m^6^A modification sites and their frequencies are mapped to their nucleotide position in the viral genome. Sites are depicted relative to their location in the HIV-1 sequence of J-Lat 10.6 cells from GenBank: MN989412.1. (**B**) Distribution of m^6^A sites in HIV-1 RNA. No sites were mapped to the 5′ UTR, so it is not represented. (**C**) The nucleotide sequences of the m^6^A consensus motif in HIV-1 RNA and cellular RNA were identified using m^6^A-SAC-seq data.

We further identified sites of m^6^A modification in HIV-1 RNA in reactivated J-Lat 10.6 cells, discovering a total of 46 m^6^A modification sites (Fig. 6A; Table S2). The most prevalent m^6^A was in the *rev* coding region, with 97% of transcripts containing modification at a single site. Several other abundant m^6^A sites were present in *gag/pol*, *env/rev*, and the 3′ UTR (Fig. 6A). The distribution of m^6^A in the HIV-1 RNA genome is shown in Fig. 6B. Furthermore, we compared the m^6^A consensus motifs in viral and cellular RNA. We found that, while the internal consensus sequence of GAC was shared among both RNA species, there appears to be a preference for A in the terminal position of the 5-nucleotide motif that does not exist for m^6^A motifs in cellular RNA (Fig. 6C).

### Validation of m^6^A-SAC-seq results by reverse transcription quantitative PCR (RT-qPCR) and meRIP

**Fig 7:**
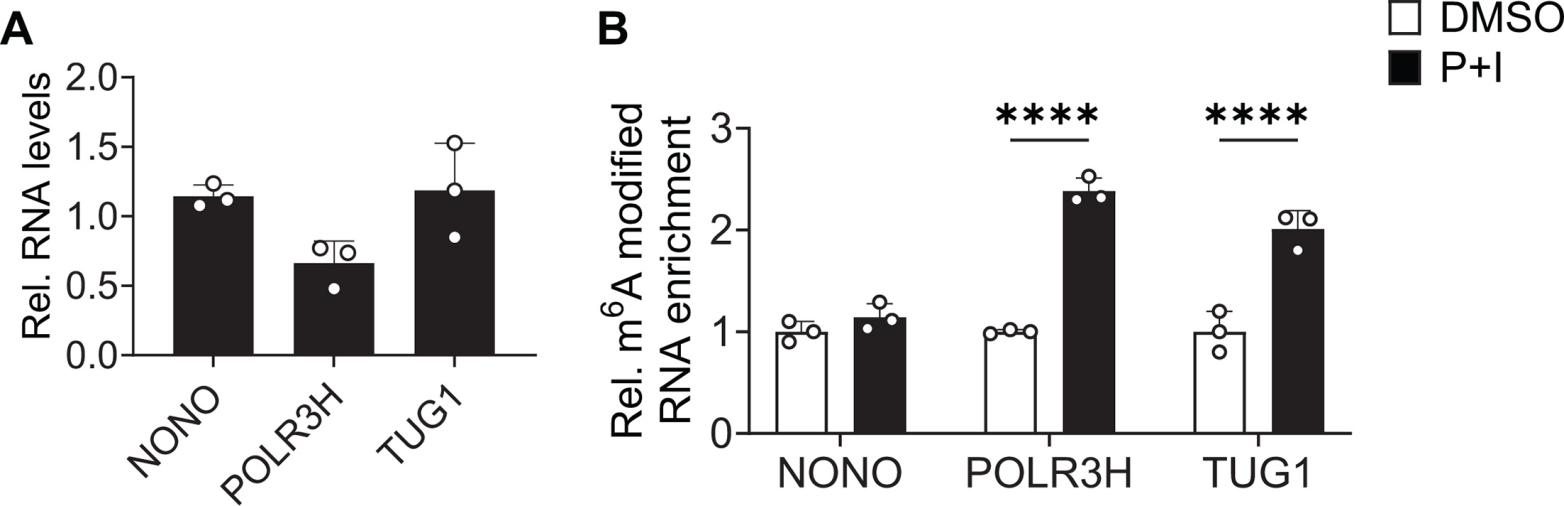
m^6^A-SAC-seq validation. (**A**) Changes in expression levels of target genes in J-Lat 10.6 cells after 6 h P + I treatment compared to DMSO treatment. RT-qPCR was performed, and qPCR data were normalized using GAPDH. (**B**) The same RNA samples used for (**A**) were subjected to m^6^A RNA-immunoprecipitation (IP). Enrichment of m^6^A-modified RNA in the IP was determined by RT-qPCR. NONO served as a negative control for m^6^A modification. Results are shown as mean ± SD from three independent experiments. Statistical analysis was performed using two-way ANOVA. ****P < 0.0001.

For the validation of our m^6^A-SAC-seq results, we chose two novel transcripts, namely, RNA polymerase III subunit H (*POLR3H*) and taurine upregulated gene 1 (*TUG1*), which shows a significant 4.5- and 4-fold increase, respectively, in the abundance of RNA m^6^A in J-Lat 10.6 cells treated with P + I compared to DMSO controls (Table S1). First, we performed RT-qPCR to assess the levels of total transcript in cells treated with DMSO or P + I. Compared to DMSO-treated cells, we observe a reduction in the levels of *POLR3H* mRNA when cells are reactivated. In contrast, there is an increase in the level of *TUG1* RNA upon P + I treatment (Fig. 7A). These results are consistent with the RNA-seq data obtained from m^6^A-SAC-seq (Table S1). We then performed meRIP to validate that m^6^A modification is increased in both *POLR3H* and *TUG1* RNA in reactivated cells. RNA from DMSO or P + I treated cells was poly(A)-enriched and then immunoprecipitated with an m^6^A-specific antibody, followed by RT-qPCR. As a negative control, we used the non-POU domain-containing octamer-binding protein (*NONO*) transcript, which is not m^6^A-modified (31) and therefore should not be enriched by meRIP (Fig. 7B). We observed a significant increase in the enrichment of *POLR3H* and *TUG1* RNA with meRIP after cells have been reactivated with P + I (Fig. 7B). These data validate the results of m^6^A-SAC-seq using independent methods. Although we have validated additional cellular genes using RT-qPCR and meRIP (data not shown), we decided to focus on *POLR3H* and *TUG1* for functional studies in HIV-1 latency reactivation.

### *POLR3H* and *TUG1* facilitate efficient HIV-1 reactivation in J-Lat 10.6 and primary T_CM_ cells

**Fig 8:**
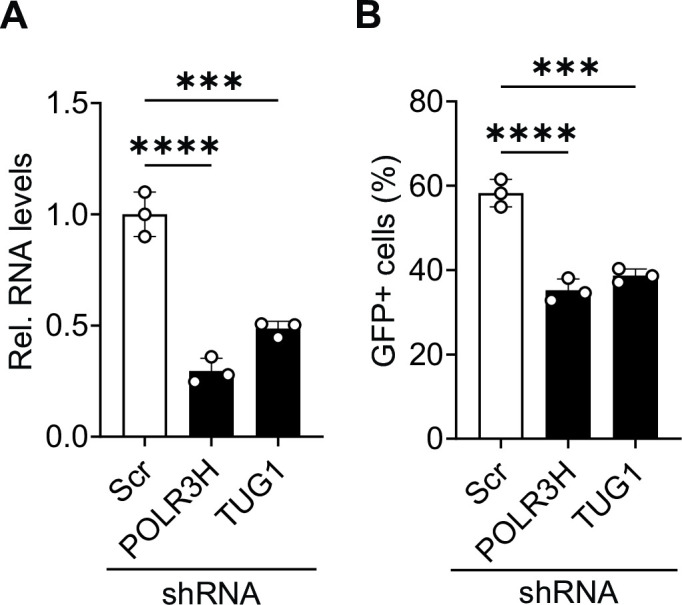
Silencing of *POLR3H* and *TUG1* impairs HIV-1 reactivation in J-Lat 10.6 cells. J-Lat 10.6 cells were transduced with a lentiviral vector that stably expresses gene-specific shRNA. Scrambled (Scr) shRNA transduced cells served as control. (**A**) Knockdown efficiency of *POLR3H* and *TUG1* mRNA was measured by RT-qPCR. (**B**) GFP-positive cells were measured after P + I treatment. Results are shown as mean ± SD from three independent experiments. Statistical analysis was performed using one-way ANOVA. ***P < 0.001 and ****P < 0.0001.

While we observed differential m^6^A modification of *POLR3H* and *TUG1* RNA in J-Lat 10.6 cells during reactivation (Fig. 7), this result did not demonstrate a role for these RNAs in the process of HIV-1 reactivation. Therefore, we next sought to determine whether *POLR3H* and *TUG1* are necessary for efficient HIV-1 reactivation in J-Lat 10.6 cells. Cells were stably transduced with lentiviral vectors expressing gene-specific short hairpin RNA (shRNA), and KD efficiency was confirmed by RT-qPCR (Fig. 8A). A non-targeting scrambled (Scr) shRNA was used as a negative control. Cells were reactivated by P + I treatment, and GFP expression was measured. We observed ~1.5-fold reduction in HIV-1 reactivation compared to control cells (Fig. 8B). These results show that KD of *POLR3H* or *TUG1* impairs HIV-1 reactivation, suggesting these transcripts have a functional role in HIV-1 reactivation.

**Fig 9:**
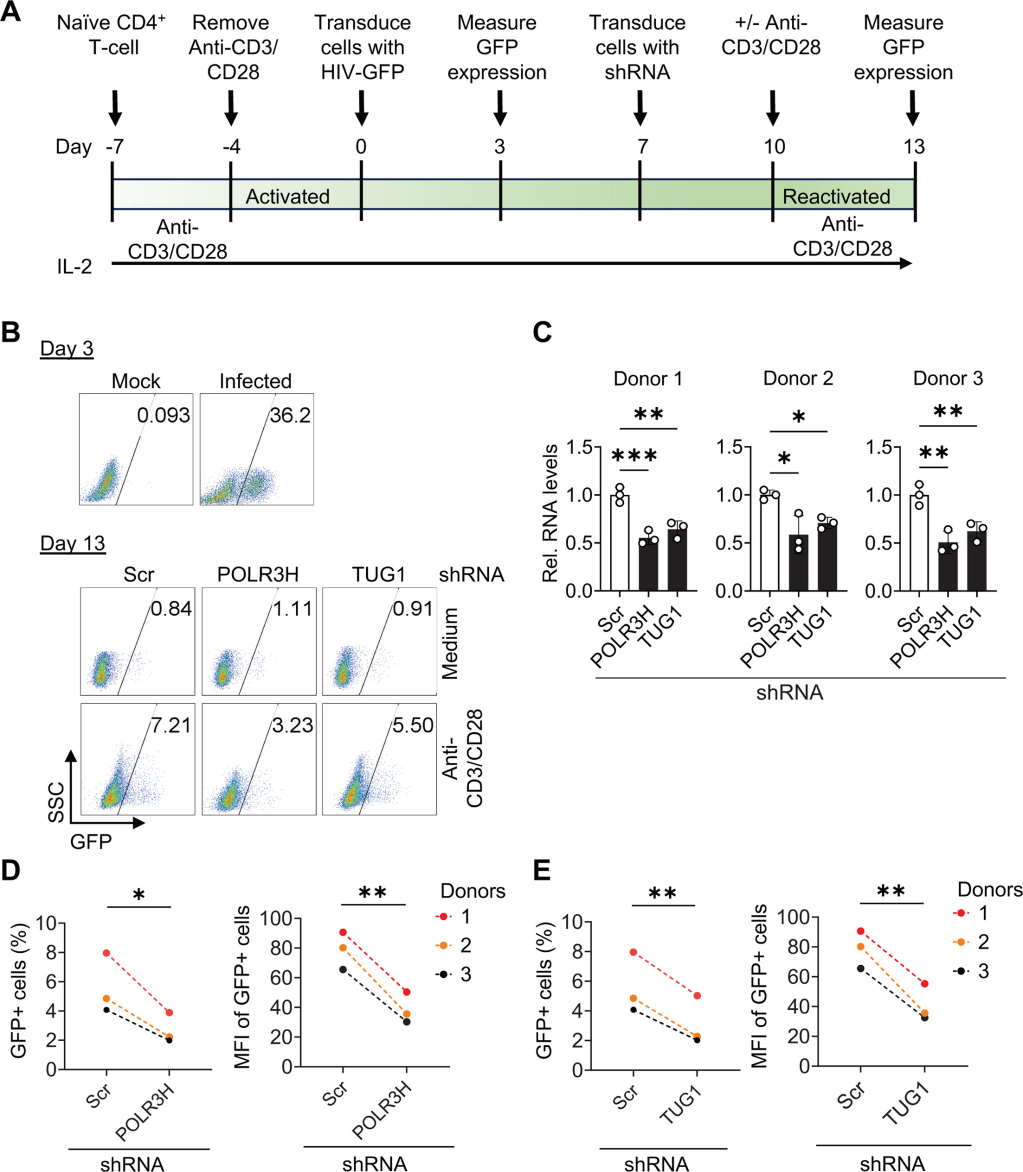
Silencing of *POLR3H* and *TUG1* impairs HIV-1 reactivation in latently infected primary T_CM_ cells. (**A**) Protocol summary. Naïve CD4^+^ T cells were isolated from PBMCs of three healthy donors. Cells were activated using anti-CD3/CD28 antibody-coated beads for 3 days and infected with vesicular stomatitis virus G protein (VSV-G)-pseudotyped HIV-1 GFP reporter to generate a primary T_CM_ model of latency. (**B**) GFP expression was measured at days 3 and 13 post-infection by flow cytometry. Representative data from a single donor are shown. SSC, side scatter. Scrambled (Scr) shRNA transduced cells served as control. (**C**) After 7 days of HIV-1 infection and culture, cells were transduced with either Scr shRNA (control) or shRNA targeting *POLR3H* or *TUG1*. Knockdown of target RNA was quantified using RT-qPCR at day 13. (**D and E**) HIV-1 reactivation was measured by GFP expression using flow cytometry at day 13. Changes in GFP percentage and MFI of GFP-positive cells from three independent donors are shown. Results from three individual donors are shown. Statistical analysis was performed using two-way ANOVA. *P < 0.05, **P < 0.01, and ***P < 0.001.

We also wanted to determine whether *POLR3H* and *TUG1* are important for reactivation of HIV-1 in latently infected primary CD4^+^ T cells. We employed an *in vitro* primary T_CM_ model of HIV-1 latency with optimized protocols (20, 21), as detailed in Fig. 9A. GFP expression was assessed at day 3 and day 13 post-infection with a GFP reporter HIV-1 vector to confirm infection and establishment of latency, respectively (Fig. 9B). At day 3 post-infection, 15%–36.2% of CD4^+^ T cells from three different donors showed GFP expression, indicating successful infection (Fig. 9B and data not shown). At day 7 post-infection, cells were transduced with lentiviral vectors expressing Scr, *POLR3H*, or *TUG1* shRNA, achieving 40%–50% KD of *POLR3H* and *TUG1* RNA expression at day 13 (Fig. 9C). Following reactivation of latently infected CD4^+^ T cells using anti-CD3/CD28 antibody-coated beads, GFP expression was quantified as a measure of HIV-1 latency reactivation 3 days post-reactivation. Medium-treated cells served as a negative control, showing basal GFP expression levels ranging from 0.8% to 1.2% (Fig. 9B and data not shown). *POLR3H* KD reduced HIV-1 reactivation by approximately twofold and *TUG1* KD reduced HIV-1 reactivation by 1.6- to 2.2-fold across three donors compared to cells transduced with Scr shRNA vector as measured by percentage and MFI of GFP-positive cells (Fig. 9D and E). These findings indicate that *POLR3H* and *TUG1* act as positive regulators of HIV-1 reactivation in latently infected primary CD4^+^ T cells.

## DISCUSSION

One of the primary challenges in curing HIV-1 infection is the persistence of proviral reservoirs in long-lived CD4^+^ T cells. Strategies to induce or prevent reactivation of HIV-1 from latency have therapeutic potential for HIV-1 cure (1). In this study, using epitranscriptomic m^6^A analysis and functional assays, we tested the hypothesis that RNA m^6^A modifications influence the efficiency of HIV-1 latency reversal.

Our data demonstrate that HIV-1 reactivation enhances cellular RNA m^6^A levels in LRA-treated J-Lat clones, which are consistent with the increase in m^6^A levels observed upon productive infection (14–17). Similar results were obtained with four different clones of J-Lat cells with different HIV-1 integration sites (32), demonstrating that this phenotype is likely due directly to the reactivation of latent HIV-1 infection and not integration-site dependent. We noticed the lack of a perfect correlation between HIV-1 activation and the increase in cellular RNA m^6^A levels in four different J-Lat cell clones (Fig. 1A through C). This observation could be explained by different efficiency of HIV-1 gene expression due to the unique integration site of the proviral DNA in the genome of each J-Lat cell clone (19, 32).

The J-Lat provirus is defective for *env* expression and virus production. Therefore, in the context of J-Lat cells, it appears that the elevation of m^6^A levels is induced by HIV-1 gene expression and does not require infectious virions or spreading infection. However, it was unclear whether elevation of m^6^A levels was a result of, or requirement for, reactivation of HIV-1 gene expression. To test this, we performed reactivation under conditions of METTL3 or METTL14 OE or KD. The results indicate that indeed, the level of reactivation in J-Lat 10.6 cells is proportional to cellular RNA m^6^A levels. METTL14 OE did not affect m^6^A or reactivation levels. These results support the notion that METTL3 is the limiting subunit for formation of the catalytically active methyltransferase complex. However, we cannot rule out that 1.4-fold upregulation of METTL14 expression might be too low to observe a phenotype on m^6^A levels (Fig. 2J through L). We further confirmed that methyltransferase activity is required for efficient latency reactivation using a METTL3-specific inhibitor (Fig. 3). Together, these findings support a positive correlation between cellular RNA m^6^A levels and HIV-1 latency reversal.

The mechanism by which HIV-1 induces upregulation of cellular RNA m^6^A levels has remained elusive. We have shown that there is no appreciable change in the levels of m^6^A writers or erasers during either productive infection (15) or reactivation of J-Lat cells (Fig. S1). Therefore, the change in m^6^A levels is not a result of higher methyltransferase complex or eraser enzymes. It is possible that HIV-1 infection alters the enzymatic activity of writers or erasers. For example, it is worth investigating whether HIV-1 reactivation affects m^6^A regulatory protein activity through posttranslational modifications (33, 34).

Earlier generations of m^6^A-seq depend on immunoprecipitation of m^6^A-modified RNA followed by high-throughput sequencing of 100–200 nt fragments, or ~20–25 nt in the case of higher resolution crosslinking immunoprecipitation coupled with sequencing (CLIP-seq) approaches (28, 35, 36). CLIP-seq has also been used to study of HIV-1 and cellular RNA binding proteins that regulate critical aspects of viral replication (37, 38). Our study utilized m^6^A-SAC-seq to analyze m^6^A modifications on HIV-1 RNA at single-nucleotide resolution (26, 27). Identification of the precise nucleotide position of m^6^A in individual transcripts allows for the design of functional studies that can yield results that are more straightforward to interpret than global KD or OE of m^6^A regulatory enzymes. Another major advantage of m^6^A-SAC-seq is that this method can be used to determine the m^6^A/A ratio at each site of modification, which allows for the determination of sites that are differentially modified under different conditions. This data set can be used to develop specific hypotheses about how regulation of m^6^A modification by HIV-1 contributes to latency reversal.

Using m^6^A-SAC-seq, we identified transcriptome-wide changes in m^6^A modifications of cellular RNA during HIV-1 latency reversal. Our analyses revealed differential methylation of 2.1% of the m^6^A content of poly(A)-enriched RNA, with 534 hypomethylated and 639 hypermethylated m^6^A sites in reactivated J-Lat 10.6 cells compared to controls (Fig. 5A). This may represent an underestimation of the actual changes in m^6^A methylation, as ~45% of J-Lat 10.6 cells remained latently infected at 6 h post P + I treatment (Fig. 1A). Our GO analysis revealed enriched cellular pathways among m^6^A hypermethylated or hypomethylated transcripts in HIV-1-reactivated J-Lat 10.6 cells, suggesting a functional link between the m^6^A profile of cellular transcripts and HIV-1 reactivation.

We identified 46 m^6^A modifications in both coding and non-coding regions of HIV-1 RNA, with AGACA as a consensus motif (Fig. 6C). This corresponds to an m^6^A content of 0.08% and is many more sites than predicted by peak calling from lower resolution sequencing approaches, which ranges from 3 to 12 sites (13, 39). Some of these sites are represented at low levels (less than 30%) and may be difficult to distinguish from background using other methods. Our study confirms the presence of m^6^A modification on a majority of HIV-1 RNAs in *gag*, *pol*, *rev*, and the 3’UTR (13, 14, 36, 40). We found that two adenosines in the 3′UTR were modified in over 50% of viral transcripts (Fig. 6A; Table S2). These m^6^A sites were also identified by direct RNA sequencing (DRS) from HIV-1 producer cells and infected cells and were shown to be involved in viral RNA splicing (39). Another m^6^A site identified in the current study and in previously published DRS data (39) is located in *env/rev* and is m^6^A modified on nearly every transcript, suggesting an important function for this modification. Further studies are necessary to reveal to the functional impact of individual m^6^A modifications of viral RNA.

We selected *POLR3H* for the validation of m^6^A-SAC-seq and to determine whether this differentially modified transcript is necessary for efficient reactivation of HIV-1 gene expression in latently infected cells. KD of *POLR3H* reduced HIV-1 latency reversal in both J-Lat 10.6 and primary T_CM_ cells. These data suggest a role for *POLR3H* in HIV-1 reactivation. Whether upregulation of m^6^A modification on the *POLR3H* transcript is also necessary for efficient HIV-1 reactivation warrants further investigation. Targeted removal of transcript and site-specific methyl groups will be necessary to determine whether individual m^6^A modifications play a role in HIV-1 reactivation. *POLR3B* is also differentially m^6^A-modified upon reactivation of J-Lat 10.6 cells (Table S1). It would be interesting to determine whether m^6^A modification of subunit RNAs ultimately affects the assembly of RNA pol III.

We also demonstrated a positive role for the long noncoding RNA (lncRNA) *TUG1* in the reactivation of J-Lat 10.6 and primary T_CM_ cells. lncRNA is known to regulate gene expression at multiple levels, including transcription, RNA processing, translation, and post-translation (41). lncRNAs regulate various aspects of HIV-1 replication and latency development (42–44). A recent study showed that treatment of human primary astrocytes with the HIV-1 Tat protein enhances lncRNA *TUG1* expression (45). Although we showed that KD of *TUG1* transcript reduces HIV-1 latency reversal, it remains to be determined how differential m^6^A modification affects the *TUG1* transcript.

Our study revealed novel m^6^A modification of HIV-1 and cellular RNA that occur in response to reactivation of J-Lat 10.6 cells. This data set will serve as a valuable resource for the broader research community for the development of hypotheses regarding the functional role of m^6^A modification in regulating gene expression during HIV-1 latency reversal.

## MATERIALS AND METHODS

### Cell culture

J-Lat cell clones (19) were obtained from NIH AIDS reagent program, Division of AIDS, NIAID, NIH. J-Lat, Jurkat, Hut/CCR5, SupT1, and primary CD4^+^ T cells were cultured in RPMI-1640 (ATCC) supplemented with 10% fetal bovine serum (FBS; R&D Systems) and antibiotics (100 U/mL penicillin and 100 µg/mL streptomycin, Gibco) as described (21, 36, 46). HEK293T cells were cultured in Dulbecco modified Eagle medium (DMEM) (Gibco) with 10% FBS and antibiotics (47). All cells were cultured at 37°C with 5% CO_2_. All cell lines tested negative for mycoplasma contamination by use of a PCR-based universal mycoplasma detection kit (ATCC 30–1012K).

### HIV-1 latency reversal in J-Lat cells and flow cytometry

J-Lat cells (5 × 10^6^ cells) were seeded 24 h before treatment with DMSO, 32 nM PMA (Sigma, P-1585) and ionomycin (1 µM) P + I (Sigma, I-0634), or 10 ng/mL TNFα (Peprotech, 300–01A) at the indicated time in figures or figure legends to induce HIV-1 gene expression (21). HIV-1 reactivation was assessed by measuring GFP expression by flow cytometry (21). GFP-based cell sorting was performed by the Flow Cytometry Facility at the University of Iowa with the Cytek Aurora CS. Flow cytometry data were analyzed by FlowJo software (47).

### Cell fractionation

Cytoplasmic and nuclear fractions were prepared using the Subcellular Protein Fractionation Kit for Cultured Cells according to the manufacturer’s instructions (ThermoFisher, 78840).

### m^6^A quantification by dot blot assay or ELISA

Dot blot analysis was performed as described (15, 22). For quantification of m^6^A levels by ELISA, a published protocol (23) was followed with modifications as described (18).

### Plasmids

pLentiCRISPR-v2 sgMETTL3 and pLentiCRISPR-v2 sgMETTL14 plasmids were described (48). lentiCRISPR v2 was from Feng Zhang (Addgene plasmid #52961) (49). To construct pLenti-Flag-METTL3-puro, Flag-METTL3 fragment from pcDNA3/Flag-METTL3 was inserted into the NdeI and XbaI sites of pLenti-puro. pcDNA3/Flag-METTL3 (Addgene plasmid #53739) and pcDNA3/Flag-METTL14 (Addgene plasmid #53740) were from Chuan He (9). pLenti-puro was from Ie-Ming Shih (Addgene plasmid #39481). pLenti-Flag-METTL14 hygro was created by inserting the Flag-METTL14 fragment from pcDNA/Flag-METTL14 into the BamHI and XhoI sites of pLenti CMV GFP Hygro. pLenti CMV Hygro (656-4) was from Eric Campeau and Paul Kaufman (Addgene plasmid #17446). pLKO.1 carrying shRNA targeting *TUG1* (5′-GCCTATGCGTTTGCGATTCGA-3′) or *POLR3H* (5′-AAACTGGAGGATGCCTATGTA-3′) were constructed by inserting annealed shRNA oligonucleotide (Integrated DNA Technologies) duplexes into the AgeI and EcoRI sites. The sequence of all constructs was confirmed by Sanger sequencing.

### Generation of stable J-Lat cell clones with altered METTL3 and METTL14 expression

J-Lat 10.6 cells were transduced with lentiviruses in the presence of polybrene (8 µg/mL) by centrifugation at 1,200 × g for 2 h at room temperature. Transduced cells were cultured in complete RPMI-1640 for 3 days prior to selection with puromycin (1 µg/mL) or hygromycin (400 µg/mL). After selection was complete, single-cell clones were obtained by limiting dilution. OE and KD of METTL3 and METTL14 were confirmed by immunoblotting (36).

### Antibodies and immunoblotting

Antibodies used for immunoblotting were METTL3 (Proteintech, 15073), METTL14 (Proteintech, 26158), FLAG (Sigma, M2), WTAP (Proteintech, 10200), ALKBH5 (Sigma, HPA007196), FTO (Abcam, Ab124892), histone deacetylase 2 (HDAC2) clone 3F3 (Cell Signaling Technologies, 5113), and glyceraldehyde-3-phosphate dehydrogenase (GAPDH; Bio-Rad, AHP1628). Immunoblotting was performed as described (46, 47).

### UZH1a treatment and cell viability assay

J-Lat 10.6 cells were seeded at a concentration of 4 × 10^5^ cells/mL, 24 h prior to treatment with DMSO or UZH1a (MedChemExpress) for 48 h. Viability was assessed by using the CellTiter 96 Non-radioactive Cell Proliferation Assay (MTT assay; Promega) as described (50).

### m^6^A-SAC-seq, data deposition, and access

m^6^A-SAC-seq was performed as previously described (26, 27). Briefly, total RNA from J-Lat 10.6 cells treated with DMSO or P + I for 6 h was isolated using TRIzol (Invitrogen). mRNA was enriched from total RNA using Dynabeads Oligo (dT)_25_. Enriched mRNA was quantified using Qubit (InvitrogenTM catalog number Q32852). Purified mRNA (150 ng) from each sample was used for m^6^A-SAC-seq.

### GO analysis

GO analysis was performed using the GO Enrichment Analysis tool (DAVID) (51, 52). GO graph was plotted using the Web server REVIGO (53).

### m^6^A immunoprecipitation and RT-qPCR

Total cellular RNA was extracted using TRIzol, and the concentration was determined with a Nanodrop-OneC spectrophotometer (Thermo Fisher). m^6^A-modified RNA was isolated as described (54, 55). RNA from the IP was used for cDNA synthesis, and RT-qPCR was performed to quantify the enrichment. Data analysis was performed using the ΔΔC_T_ method with a spike in luciferase RNA as an internal control (New England Biolabs).

### T_CM_ model of HIV-1 latency and reactivation

Healthy deidentified donor blood was purchased from the DeGowin Blood Center at the University of Iowa. PBMCs were isolated from healthy donor blood as described (18, 54). Naïve CD4^+^ T cells were enriched using EasySep Human CD4^+^ T cells isolation kit (STEMCELL Technologies) and cultured in RPMI-1640 to generate the T_CM_ model of HIV-1 latency as described (20, 21). Briefly, naïve CD4^+^ T cells were stimulated using CD3/CD28 beads (Dynabeads Human T-activator CD3/CD28) for 3 days. After 4 days of additional culture, primary CD4^+^ T cells (1 × 10^6^) were infected with single-cycle HIV-1 GFP reporter (NL4-3ΔEnv-GFP) pseudotyped with vesicular stomatitis virus G protein and then cultured for additional 7 days to produce latently infected T_CM_ cells. Subsequently, infected cells were transduced with Scr shRNA, shRNA *TUG1*, and shRNA *POLR3H* for 3 days before activating with anti-CD3/CD28 antibody T cell activator solution (ImmunoCult, STEMCELL technologies) for additional 3 days. At day 13, *TUG1* and *POLR3H* RNA expression normalized to *GAPDH* was determined by RT-qPCR. HIV-1 reactivation was measured in mock and anti-CD3/CD28 treated cells by flow cytometry (21).

### Oligonucleotide sequences

Oligonucleotide sequences used for qPCR amplification were:

NONO forward 5′ CCGACATCACTGAGGAAGAAA 3′

NONO reverse 5′ CCAAGCGGATAAAGCCAAATC 3′

POLR3H forward 5′ GGAAATGGTGGACACCGTCC 3′

POLR3H reverse 5′ CAGAGTCCCACGTTGTACAC 3′

TUG1 forward 5′ GTGCAGAAGCCCAGAGTAAA 3′

TUG1 reverse 5′ CCACGGTGGTAAAGGAAGATAG 3′

GAPDH forward 5′ GGAAGGTGAAGGTCGGAGTCAACGG 3′

GAPDH reverse 5′ CTGTTGTCATACTTCTCATGGTTCAC 3′

### Statistical analysis

Data were analyzed using either unpaired *t*-test or one- or two-way analysis of variance with Prism software, and statistical significance was defined as *P* < 0.05.

## Data Availability

The m^6^A-SAC-seq data have been deposited in the Gene Expression Omnibus (GEO) with accession number GSE273614.

## SUPPLEMENTAL MATERIAL

Supplemental material mbio.02214-24-s0001.pdf Fig. S1 and captions for Tables S1 and S2.

Table S1 mbio.02214-24-s0002.xlsx m^6^A-SAC-seq and RNA-seq data for J-Lat 10.6 cells.

Table S2 mbio.02214-24-s0003.xlsx m^6^A-SAC-seq identifies m^6^A modification sites in HIV-1 RNA in reactivated J-Lat 10.6 cells.

ASM does not own the copyrights to Supplemental Material that may be linked to, or accessed through, an article. The authors have granted ASM a non-exclusive, world-wide license to publish the Supplemental Material files. Please contact the corresponding author directly for reuse.
