# Supplementary material for: Epitranscriptomic m6A modifications during reactivation of HIV-1 latency in CD4+ T cells
Source: mBio. 2024 Oct 7;15(11):e02214-24. doi: 10.1128/mbio.02214-24 (PMC11559067; doi:10.1128/mbio.02214-24)
Supplement: Supplemental material — Fig. S1 and captions for Tables S1 and S2. [file mbio.02214-24-s0001.pdf]

Supplemental information

Epitranscriptomic m^{6}A Modifications During Reactivation of HIV-1 Latency in CD4$^+$ T Cells

Tarun Mishra,[1] Stacia Phillips,[1, *] Yutao Zhao,[2, *] Bethany Wilms,[1] Chuan He,[2,3] Li Wu,[1,#]

[1] Department of Microbiology and Immunology, Carver College of Medicine, The University of Iowa, Iowa City, Iowa, USA

[2] Department of Chemistry, Department of Biochemistry and Molecular Biology, Institute for Biophysical Dynamics, University of Chicago, Chicago, Illinois, USA

[3] Howard Hughes Medical Institute, University of Chicago, Chicago, Illinois, USA

[*] Stacia Phillips and Yutao Zhao contributed equally to this article.

[#] Address correspondence to Li Wu, li-wu@uiowa.edu

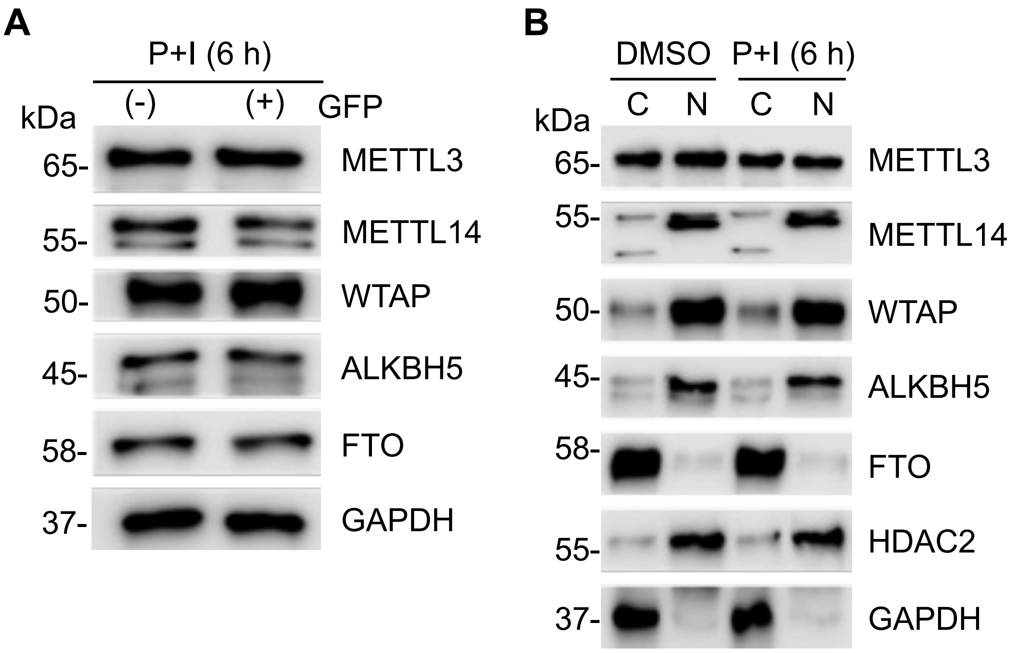

Figure S1. Reactivation of J-Lat 10.6 cells does not affect the expression or nucleocytoplasmic localization of m^{6}A regulatory proteins. (A) J-Lat 10.6 cells were treated with PMA and ionomycin (P+I) for 6 h and then sorted by flow cytometry based on GFP positivity (see Fig. 4D). The levels of m^{6}A writers (METTL3, METTL14, and WTAP) and erasers (ALKBH5 and FTO) were determined by immunoblot using whole cell lysates. **(B)** Unsorted J-Lat 10.6 cells treated with DMSO as a negative control or P+I were subjected to nucleocytoplasmic fractionation and the levels of the m^{6}A writer complex (METTL3, METTL14, and WTAP) and erasers (ALKBH5 and FTO) were determined by immunoblot using lysates from the separated fractions. HDAC2 and GAPDH serve as markers of successful separation of the nuclear (N) and cytoplasmic (C) fractions, respectively.

Supplemental table information

Table S1. m^6A-SAC-seq and RNA-seq data of J-Lat 10.6 cells. Cells were treated with P+I or DMSO control for 6 h and poly(A)-enriched RNA was analyzed based on three biological replicates. m^6A-SAC-seq and RNA-seq data are in two separate sheets in one Excel file. Data of *POLR3H* or *TUG1* genes are highlighted in yellow.

Table S2. m^6A-SAC-seq identifies m^6A modification sites in HIV-1 RNA in reactivated J-Lat 10.6 cells. Cells were treated with P+I or DMSO control for 6 h and poly(A)-enriched RNA was analyzed based on three biological replicates. m^6A motifs, m^6A/A ratio of individual samples and their average values are included in the Excel file.
